# Interrogation of the Pathogen Box reveals small molecule ligands against the mycobacterial trehalose transporter LpqY-SugABC†

**DOI:** 10.1039/d2md00104g

**Published:** 2022-08-16

**Authors:** Anjana Radhakrishnan, Chelsea M. Brown, Collette S. Guy, Charlotte Cooper, Raul Pacheco-Gomez, Phillip J. Stansfeld, Elizabeth Fullam

**Affiliations:** School of Life Sciences, University of Warwick Coventry CV4 7AL UK e.fullam@warwick.ac.uk +44 (0)2476 574239; Department of Chemistry, University of Warwick Coventry CV4 7AL UK; Malvern Panalytical Ltd, Enigma Business Park Grovewood Road Malvern WR14 1XZ UK

## Abstract

Tuberculosis, caused by *Mycobacterium tuberculosis*, claims ∼1.5 million lives annually. Effective chemotherapy is essential to control TB, however the emergence of drug-resistant strains of TB have seriously threatened global attempts to control and eradicate this deadly pathogen. Trehalose recycling *via* the LpqY-SugABC importer is essential for the virulence and survival of *Mtb* and inhibiting or hijacking this transport system is an attractive approach for the development of novel anti-tubercular and diagnostic agents. Therefore, we interrogated the drug-like compounds in the open-source Medicines for Malaria Pathogen Box and successfully identified seven compounds from the TB, kinetoplastids and reference compound disease sets that recognise LpqY. The molecules have diverse chemical scaffolds, are not specific trehalose analogues, and may be used as novel templates to facilitate the development of therapeutics that kill *Mtb* with a novel mechanism of action *via* the mycobacterial trehalose LpqY-SugABC transport system.

## Abstract 2

Interrogation of the Pathogen Box identified diverse chemical scaffolds against the mycobacterial trehalose transporter.

## Graphical abstract

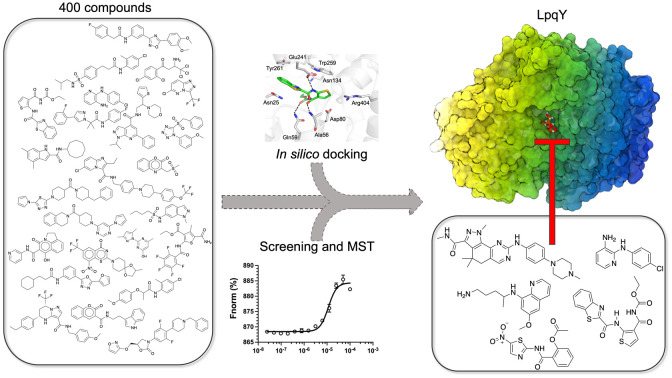

## Introduction

Tuberculosis (TB), caused by *Mycobacterium tuberculosis* (*Mtb*) is often regarded as an ancient disease and yet even in the 21st century TB still remains an urgent global health challenge. According to the latest figures from the World Health Organisation (WHO), TB is the leading cause of death from a single bacterial pathogen with ∼10 million new cases of TB infection and an estimated death toll of ∼1.5 million in 2020 alone.^1^ Despite the availability of therapeutic agents to effectively treat drug-sensitive TB infections, the emergence of drug-resistant strains of *Mtb* with low cure rates have seriously threatened the world-wide control of TB.^2^ To eradicate drug-resistant strains and eliminate TB infections we urgently need new drugs and effective therapeutic options that kill *Mtb* with novel mechanisms of action.

One of the significant challenges in TB drug discovery efforts is that the highly complex and ‘waxy’ *Mtb* cell envelope provides an intrinsic permeability barrier that prevents many potential antibiotics from gaining access to the required cytoplasmic target.^3,4^ Therefore, new initiatives are underway to develop strategies that kill *Mtb* by circumventing the need for a chemotherapeutic agent to cross the cell envelope barrier. This includes the development of molecules and aptamers that directly target structural components of the mycobacterial cell envelope,^5–9^ inhibitors of proteins localised in either the cell envelope or periplasmic space with greater accessibility to antibiotics^10,11^ and agents which perturb the inner mycobacterial membrane.^12,13^ An alternative approach is to use the endogenous *Mtb* transporters to enable the efficacious uptake of pathogen specific bactericidal agents.^14,15^ The *Mtb* genome encodes for at least 37 ATP-binding cassette (ABC) transporters and 30 major facilitator permeases,^16–19^ and the functional role of these import systems is beginning to emerge with a diverse range of substrates identified such as sugars, lipid head-groups, vitamins, ions and amino acids.^20–27^ As well as playing an important role in the control and assimilation of an array of metabolites from within a nutrient deprived host-niche environment, many *Mtb* transport systems are also implicated in pathogenicity and survival ability.^23,25,27,28^ This suggests that these import systems are important and active during infection and could be inhibited directly, or exploited to enable uptake of anti-mycobacterial agents across the cell envelope.

The *Mtb* LpqY-SugABC sugar transporter has recently received attention as an attractive target for the development of both therapeutic and diagnostic agents and, crucially, lacks an obvious homologue in humans.^23^ LpqY-SugABC salvages the non-mammalian disaccharide trehalose (α-d-glucopyranosyl-α-d-glucopyranoside, α,α-trehalose) released from the abundant trehalose containing glycolipids of the mycobacterial cell envelope.^23^ This recycling pathway plays an important role in the virulence and survival of *Mtb* in mice as demonstrated by attenuation of infection^23^ and enhanced susceptibility to antibiotics of the *Mtb lpqy-sugABC* deletion mutant *in vitro*.^29^ A diverse range of unnatural modified trehalose analogues are recognised by LpqY-SugABC and readily taken up by this transporter, suggesting that this import system has the potential to transport alternate substrates.^21,30–34^ Understanding the structural requirements for compounds to be substrates for LpqY-SugABC will provide a framework to influence drug design, either for direct targeting to dysregulate trehalose assimilation or as a route to facilitate transport of bactericidal agents.

As part of an initiative directed towards the discovery of molecules that either inhibit or hijack the LpqY-SugABC transporter, we interrogated the open-access Pathogen Box released by the Medicines for Malaria Venture. The box contains 400 chemically diverse drug-like molecules with known biological activity against a range of neglected tropical diseases, with 30% active against *Mtb*.^35,36^ The compound set has acceptable oral absorption and low cytotoxicity profiles and has formed the basis of many screening programs to facilitate the identification of new chemical starting points in drug discovery campaigns.^37–39^

In this study we screened the entire Pathogen Box compound library for *in vitro* binding to the mycobacterial LpqY substrate binding domain. This screen led to the discovery of 7 molecules with microscale thermophoresis binding scores for LpqY >12. The top hit MMV090930 is the small molecule TCA1, which has potent activity against drug-susceptible and drug-resistant strains of *Mtb* and targets the decaprenyl-phosphoryl-β-d-ribofuranose oxidoreductase DprE1 to block cell-wall arabinan biosynthesis.^40,41^ Four additional compounds with binding scores >12, which were commercially available, were selected for further evaluation, three of which were known to have antimycobacterial activity and another novel hit with inhibitory activity in kinetoplastids. Biochemical, biophysical and *in silico* approaches were employed to gain insights into LpqY-compound interactions. This study provides the first report of molecules targeting LpqY that are not specific trehalose analogues and highlights the potential for compounds outside the umbrella of ‘anti-TB molecules’ for use in targeting the LpqY-SugABC transporter. These hit compounds may be used as new chemical scaffolds or templates for the development of molecules that can target or be imported by the *Mtb* trehalose transport system.

## Methods

### Materials and reagents

All chemicals and reagents were purchased from Merck or Carbosynth. The hit compounds were purchased as follows: primaquine (Carbosynth), nitazoxanide (Carbosynth), milciclib (Stratech Scientific), TCA1 (Life Chemicals), MMV676524 (Merck).

### Pathogen Box®

The Pathogen Box (400 compounds) was provided by the Medicines for Malaria Venture (MMV, Geneva, Switzerland). The compounds were supplied as 10 mM stocks in DMSO (10 μL each) in 96-well plates. To avoid multiple freeze–thaw cycles, the supplied master plates were diluted to a concentration of 1 mM in DMSO, aliquoted and stored in 96-well plates at −80 °C.

### Protein expression and purification of *Mycobacterium thermoresistible* (*Mtr*) LpqY

The LpqY protein was produced and purified as previously described.^21^*Escherichia coli* BL21(DE3) transformed with the *mtr_lpqY_sumo* plasmid was grown at 27 °C to an optical density at 600 nm (OD_600_) of 0.6–0.8 in terrific broth supplemented with 50 μg mL^−1^ kanamycin. Protein production was induced with 1 mM isopropyl-β-thiogalactopyranoside (IPTG) and the cultures grown at 16 °C overnight. The cells were harvested by centrifugation and resuspended in lysis buffer (20 mM Tris, 300 mM NaCl, 10% glycerol, pH 7.5 (buffer A)) supplemented with a cOmplete™ protease inhibitor tablet (Roche), 5 mM MgCl_2_, 2 mg of DNase, and 40 mg of lysozyme and sonicated. Following centrifugation, the supernatant was filtered and loaded onto a pre-equilibrated HisPur Ni^2+^-NTA affinity resin (Thermo Scientific). The column was washed with buffer A and *Mtr* LpqY protein was eluted from the Ni^2+^ resin with increasing concentrations of imidazole. Fractions containing *Mtr* LpqY were digested with His-tagged SUMO protease and dialyzed against buffer A and purified with a second Ni^2+^-NTA column. The elutions containing *Mtr* LpqY were pooled and purified further using size exclusion chromatography (HiLoad 16/600 Superdex 200, GE Healthcare) and *Mtr* LpqY fractions were combined. Purified *Mtr* LpqY was concentrated to 5–10 mg ml^−1^ (Vivaspin 20; GE Healthcare) and stored at −80 °C. For microscale thermophoresis assays (MST) the purified *Mtr* LpqY protein (6 μM, in 50 mM HEPES, 300 mM NaCl, pH 7.5) was labelled with the amine reactive RED-NHS 2nd generation dye (6 μM) (NanoTemper Technologies) prior to freezing. Unreacted excess RED-NHS dye was removed on a desalting column (Zeba Spin Desalting column (7K MWCO) (ThermoScientific)), the protein concentration adjusted to 1 μM and the labelled LpqY protein was snap-frozen in liquid nitrogen and stored at −80 °C.

### Single dose screening of the Pathogen Box

For the initial screen, the 400 compounds were diluted in 96-well plates to a final concentration of 200 μM in PBS containing 0.05% Tween 20 (PBST) and evaluated for binding against LpqY by using a binding check microscale thermophoresis assay. The final concentration of LpqY in the assay was 500 nM and the final concentration of compound was 100 μM (10% final DMSO concentration). The MST screening was performed on a Monolith NT.115 instrument (NanoTemper Technologies) at 21 °C. Medium infrared laser MST power, LED light at 20% with laser off/on times of 0 s and 20 s parameters used in all MST experiments. The compound screen was validated using trehalose as a control substrate, and all compounds compared to a reference of LpqY with the addition of buffer (PBST, 10% final concentration DMSO). A total of 8 samples, in duplicate, were analysed for each MST run, which included the LpqY reference sample (buffer control), LpqY control with trehalose (100 μM) and 6 compounds from the Pathogen Box. The samples were loaded into standard treated capillaries and incubated for 10 min before analysis. Each capillary scan was evaluated to determine if the initial fluorescence values were within ±20% of the reference sample and for any aggregation. The MST response was determined for each compound (MO.Control Software, (version 1.6.1)) and the results were exported. The MST signal to noise ratio (S/N) was calculated for each compound relative to the reference using the following equation:
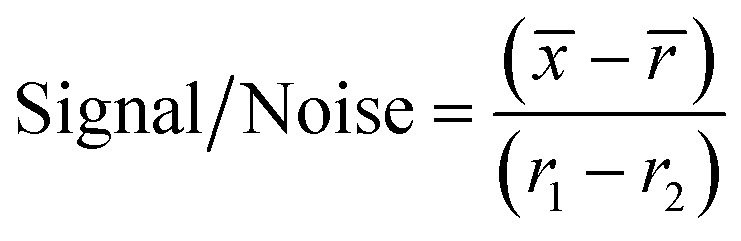
*x̄* is the mean MST response of the sample capillaries (LpqY plus compound or trehalose); *r̄* is mean MST response of the reference capillaries (LpqY plus buffer); *r*_1_ is the MST response of reference sample 1; *r*_2_ is the MST response of reference sample 2.

Samples with a signal to noise threshold >5 were identified as hits and were re-evaluated by MST with quadruplicate replicates and a separate batch of purified LpqY as a biological replicate: 4 reference capillaries (LpqY plus buffer) and 4 sample capillaries (LpqY plus compound). The binding scores were calculated directly from the MO.Control software (version 1.6.1) using the following equation:
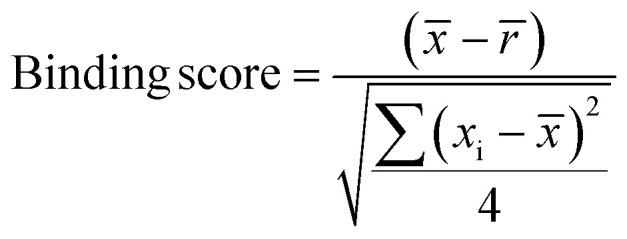
*x̄* is the mean MST response of the sample capillaries (LpqY plus compound or trehalose); *r̄* is the mean MST response of the reference capillaries (LpqY plus buffer), *x*_i_ is the individual MST response value for each sample (LpqY plus compound or trehalose).

### Affinity studies with microscale thermophoresis (MST)

Compound stocks of trehalose (20 mM), TCA1 (0.2 mM), nitazoxanide (2 mM), milciclib (10 mM), MMV676524 (4 mM) and primaquine (100 mM) were prepared in PBST containing 20% DMSO, with the exception of milciclib which was prepared in PBST containing 40% DMSO. Serial two-fold dilutions in PBST containing 20% DMSO, or 40% DMSO for milciclib, were prepared. Each compound concentration was added to RED-NHS labelled LpqY (100–500 nM) in a 1 : 1 ratio, to give a final DMSO concentration of 10% for each assay apart from milciclib (20% DMSO). The protein and compounds were incubated for 10 min at room temperature before analysis by MST, using the settings described above. All experiments were carried out in triplicate. The binding affinities were calculated using a single-site binding model within the MST NT Analysis software, MO.Affinity Analysis (version 7.0) with the following equation:
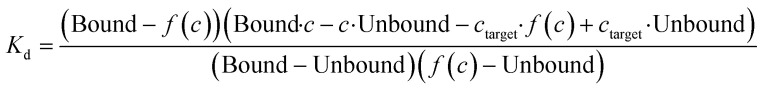

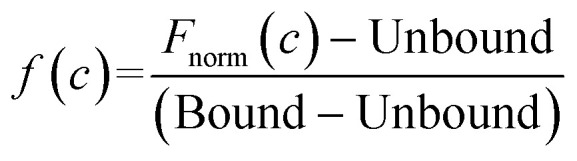
*K*_d_ is the binding affinity; Bound is the *F*_norm_ signal of the LpqY-ligand complex; Unbound is the *F*_norm_ signal of LpqY alone; *c* is the ligand concentration; *f*(*c*) is the fraction bound at a given ligand concentration; *c*_target_ is the final concentration of target in the assay; *F*_norm_(*c*) is the *F*_norm_ signal at a given ligand concentration (*c*); *F*_norm_ is the normalised fluoresence signal.

### *In silico* docking

The five compounds that were available commercially, along with trehalose, were prepared for docking using AutoDock Tools (ADT).^42^ The structures of the ligands were downloaded in .sdf file format from PubChem^43^ and converted to .mol2 file using Avogadro.^44^ The ligands were prepared with bond lengths optimised and hydrogens added. The receptor was prepared using the *Mtr* LpqY crystal structure (PDB 7APE), with ADT used to optimise bonds lengths and add hydrogens. AutoDock Vina^45^ was used to dock the flexible ligands to the rigid protein. A grid size of 30 × 30 × 30 *xyz* was used, with an exhaustiveness of 32. Each ligand was docked independently in three repeats and the pose with the most favourable energy change used for comparison. The ligand and binding site were then energy minimised using Maestro (Schrödinger release 2022-1: Maestro, Schrödinger, LLC, New York, NY, 2021.) to optimise the interactions. The docking results were visualised in PyMOL (The PyMOL Molecular Graphics System, version 2.0 Schrödinger, LLC).

## Results and discussion

### Screening the Pathogen Box against LpqY

**Fig. 1:**
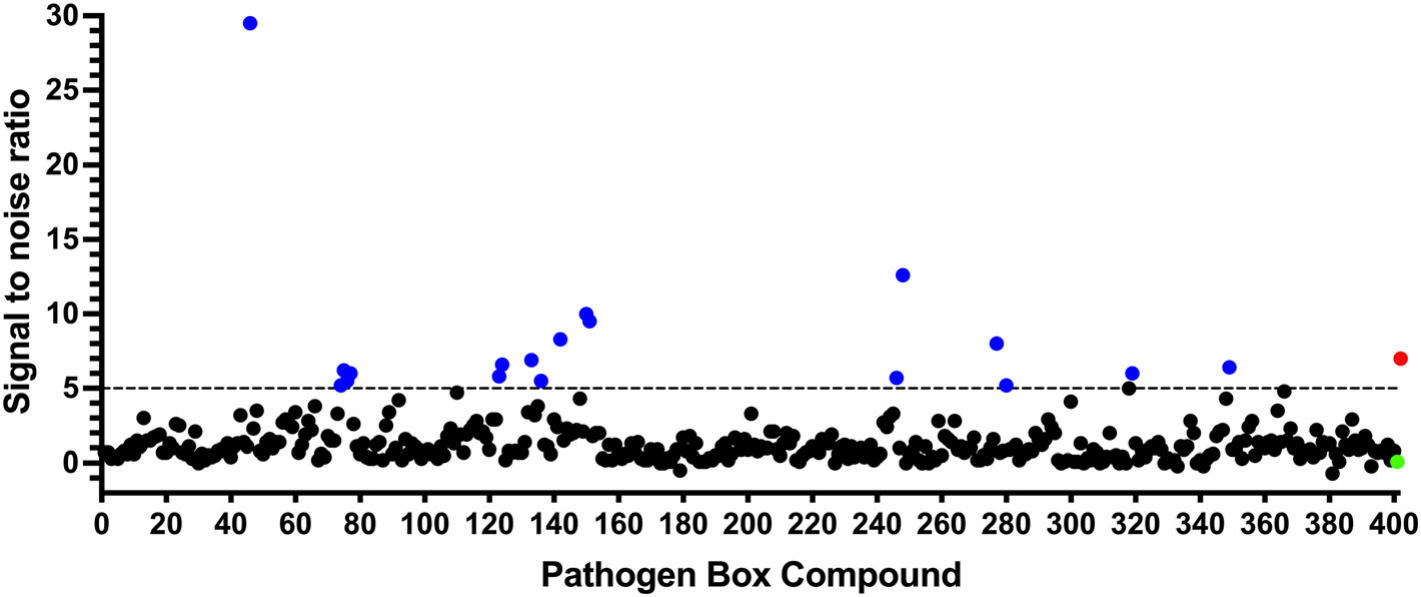
Screening of the Pathogen Box library. The Pathogen Box compounds were screened against LpqY at a single concentration of 100 μM. The signal to noise (S/N) threshold >5 is indicated with the dotted line. Compounds with a S/N ratio >5 were designated as hits and represented with blue dots; the green dot is the buffer only control and the red dot is trehalose. The identities of each compound and the associated S/N ratio are listed in ESI† List S1.

To identify compounds which interact with the LpqY substrate binding domain of the mycobacterial trehalose transporter system we purified the *Mycobacterium thermoresistible* LpqY protein, with high sequence identity to the *Mtb* LpqY homologue, and optimised a microscale thermophoresis (MST) screening assay to test all 400 Pathogen Box compounds, in duplicate, at a single concentration of 100 μM (Fig. 1). Trehalose, which gave a signal to noise (S/N) ratio of 6.7, was used as a positive control. Positive hits were defined as compounds that gave a S/N ratio >5, resulting in the identification of a total of 18 compounds with affinity to LpqY, Fig. 1, Table S1.† The identities of each compound and the associated binding score are listed in ESI† List S1.

**Fig. 2:**
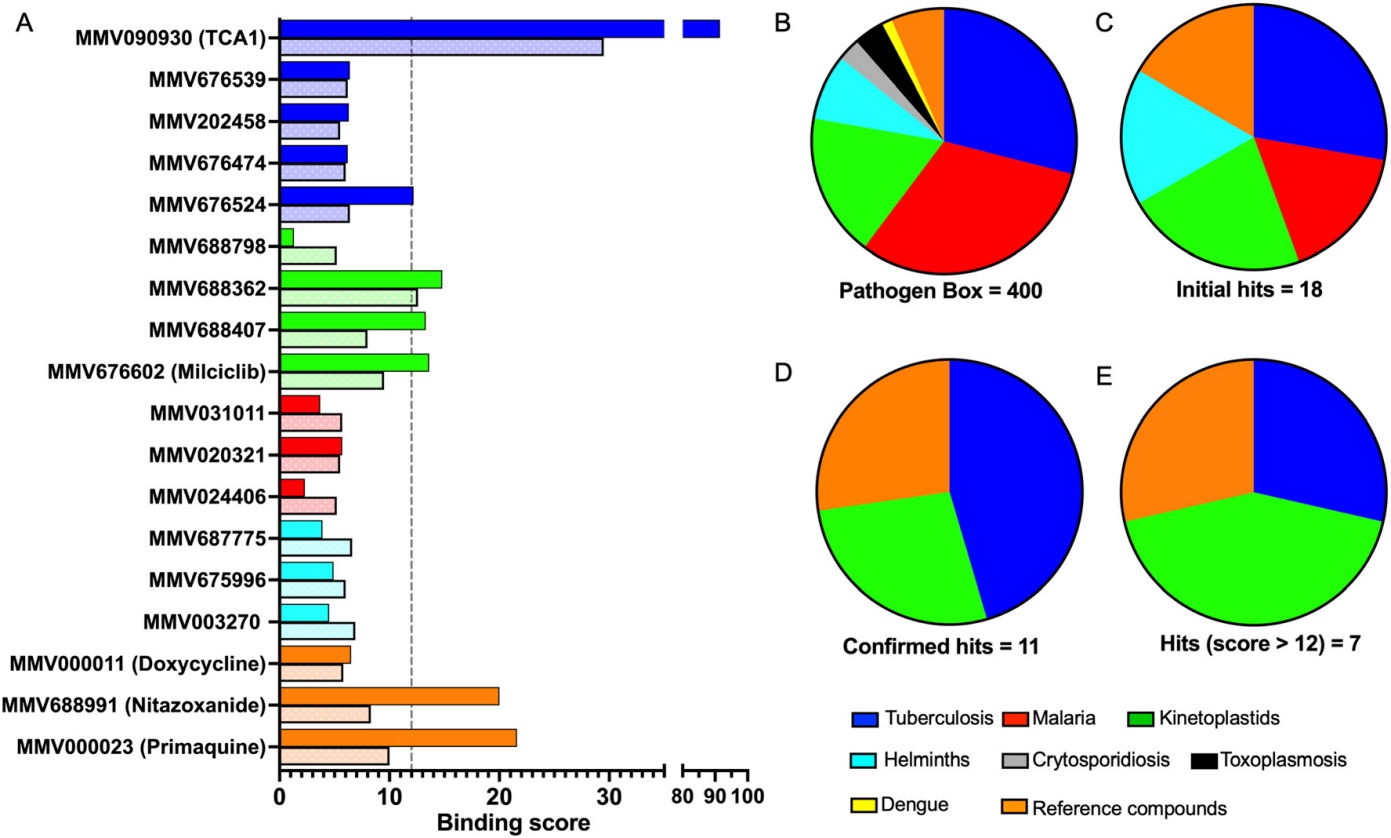
Pathogen Box compounds selected as hits for LpqY. A) Microscale thermophoresis binding scores of the hit compounds (dark colour) compared to the signal to noise scores from the initial screen (light colour). The compounds are coloured by disease area: tuberculosis (blue), kinetoplastids (green), malaria (red), helminths (cyan), reference compounds (orange). B–E Comparison of the compounds by disease area: B) Pathogen Box distribution. C) Initial hits with a signal to noise ratio >5, D) validated hits with a binding score >5, E) hits with a binding score >12.

**Fig. 3:**
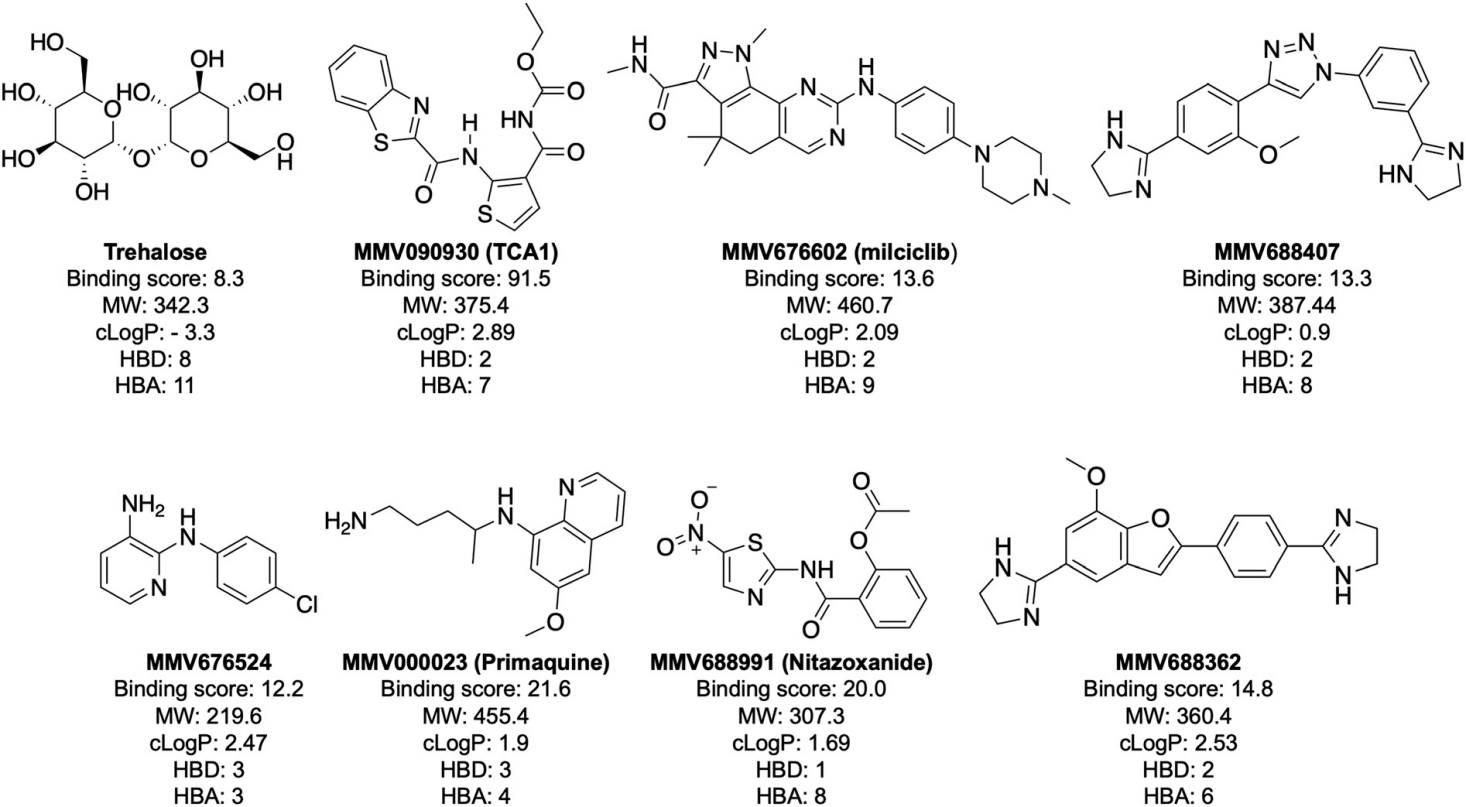
Structure and physicochemical properties of compounds with a binding score to LpqY >12. MW is molecular weight (Da). The partition coefficient (clog *P*) values for the hit compounds are from the information provided by MMV for the Pathogen Box. The clog *P* value for trehalose was calculated using ChemDraw software. HBD: hydrogen bond donor; HBA: hydrogen bond acceptor. HBD and HBA were calculated with the Molinspirations property calculations service (https://www.molinspiration.com).

In order to confirm binding and eliminate any false-positives, the 18 hit compounds with a S/N ratio >5 were re-screened against a new batch of LpqY in quadruplicates, using more stringent selection criteria and the noise calculated from the standard deviation of the four replicates. Importantly, no fluorescence effects, aggregation or adsorption were observed. This confirmed 11 hit compounds interacting with LpqY with a binding score >5, translating to a hit rate of 2.8%, and includes the three reference compounds doxycycline, primaquine and nitazoxanide, (Table S1†). Three hits: MMV676602 (milciclib), MMV688362, MMV688407 were from the disease set annotated as anti-kinetoplastids and, interestingly, an over-representation of compounds classified with known anti-tubercular activity: MMV090930 (TCA1), MMV202458, MMV676539, MMV676474, MMV676524 were identified (Fig. 2). No compounds from the malaria, toxoplasmosis, cryptosporidiosis or dengue groupings were recognised by LpqY under these assay conditions. Amongst the confirmed hits, 7 compounds had high binding scores >12. These top hits were distributed between the reference compounds (nitazoxanide and primaquine), the kinetoplastids (milciclib, MMV688362, MMV688407) and the TB disease set (TCA1, MMV676524) (Fig. 2 and 3).

Notably, the 7 hit compounds are structurally diverse, lack common structural features and have varied physiochemical properties suggesting that LpqY can recognise a broad range of chemical scaffolds (Fig. 3). It is particularly interesting that this screen identified the thiophene anti-tubercular agent TCA1, which is known to inhibit the vulnerable decaprenylphosphoryl-β-d-ribose 2′-epimerase (DprE1) enzyme,^40,41,46^ however no target has been suggested for MMV676524. DprE1 was identified as the target of TCA1 through sequencing TCA1 resistant mutants,^41^ however the compound was still active in a DprE1 overexpression strain, implying additional targets for this molecule. Indeed MoeW, involved in molybdenum cofactor biosynthesis, was identified from affinity pull-down experiments as a secondary target.^41^ As LpqY is not essential *in vitro* and is membrane-associated it is possible that LpqY may have been missed by these conventional genetic and affinity-based approaches. Whilst two of the hits, nitazoxanide and primaquine, belong to the reference compound set, they do exhibit anti-tubercular activity but lack defined targets.^47–49^ Nitazoxanide is an antiparasitic drug identified as active against replicating and non-replicating *Mtb* and has since been shown to interfere with intra-bacterial pH by disrupting membrane potential.^47,50,51^ Primaquine is an antimalarial compound that exhibits anti-mycobacterial activity *in vitro*, and inhibits growth in an intracellular macrophage model of infection.^51^ There have been no reported studies exploring the anti-mycobacterial activity of the anti-kinetoplastid compounds milciclib, MMV688362 and MMV688407.

### Determining the binding affinity and selectivity towards LpqY of the hit compounds

**Fig. 4:**
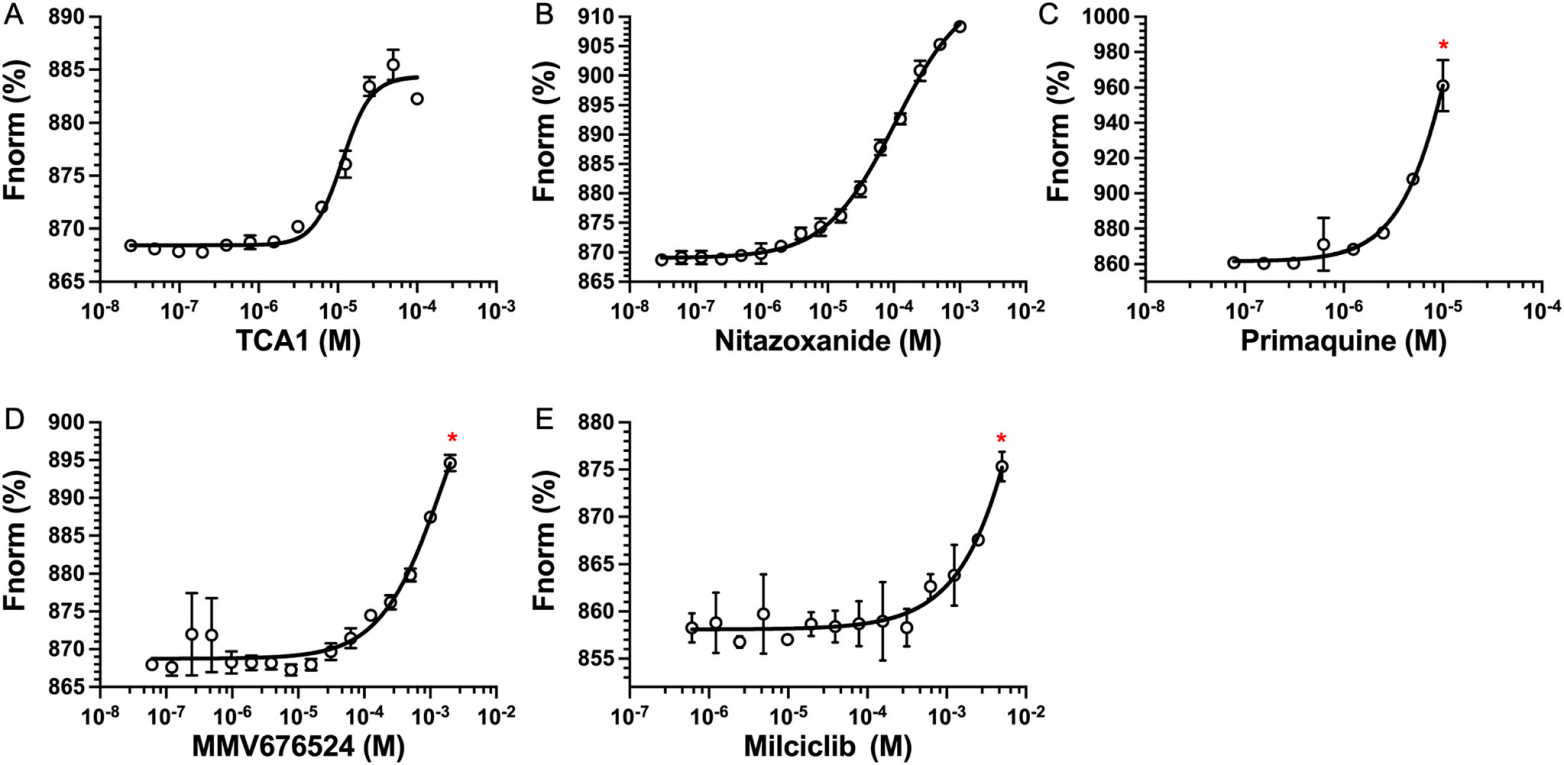
Binding affinities for LpqY. A) TCA1 (*K*_d_ 14.2 ± 0.8 μM), B) nitazoxanide (*K*_d_ 85.4 ± 3.4 μM), C) primaquine, D) MMV676524), E) milciclib. *K*_d_ measured by microscale thermophoresis (MST). *F*_norm_ (%) is the normalised fluorescence signal of the change in MST signal. Error bars represent standard deviations from at least three independent experiments. * Denotes the maximum concentration of compound tolerated in the assay. Trehalose (*K*_d_ 36.1 ± 0.5 μM, Fig. S1†).

The top five hits that were commercially available were selected for further characterisation and the binding affinities determined with LpqY (Fig. 4, Table S2†). The MST assay indicated that TCA1 has an ∼2.5-fold increase of affinity (*K*_d_) for TCA1 compared to trehalose (*K*_d_ 36.1 μM) with a *K*_d_ of 14.1 μM, which is consistent with the high binding score of 91.5 observed in the binding assay (Fig. 2). In contrast, nitazoxanide, which had a binding score of 20.0, had an ∼2-fold-decrease in affinity with a *K*_d_ of 85.4 μM. Concentration dependent binding of primaquine, MMV676524 and milciclib to LpqY was observed under these assay conditions (Fig. 4). However, the binding affinities could not be determined as high concentrations of primaquine induced fluorescent quenching of labelled LpqY and MMV676524 and milciclib have poor solubility in aqueous buffer.

**Fig. 5:**
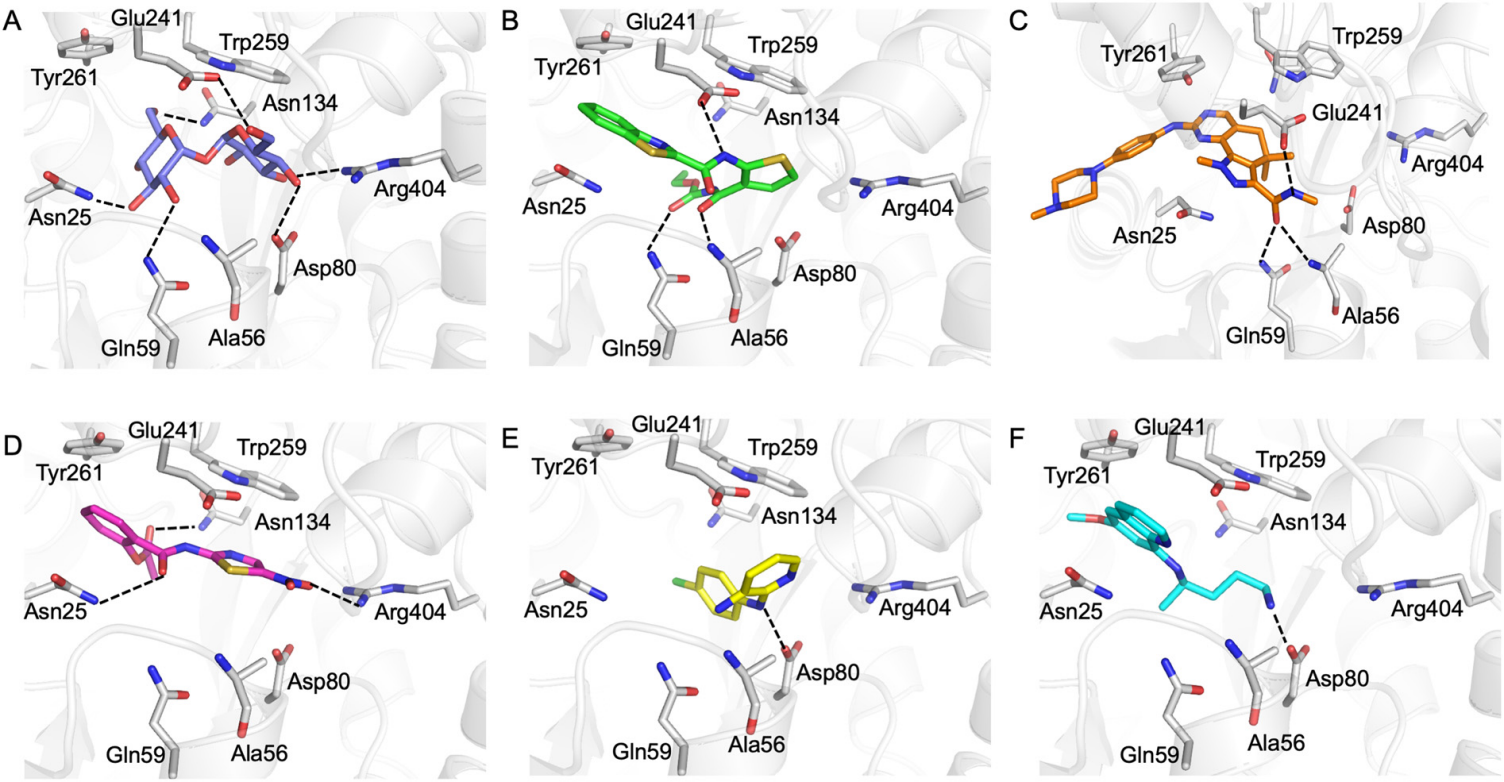
*In silico* analysis of Pathogen Box hit compounds with LpqY. A) Trehalose (blue carbon atoms), B) TCA1 (green carbon atoms), C) milciclib (orange carbon atoms) D) nitazoxanide (magenta carbon atoms), E) MMV676524 (yellow carbon atoms), F) primaquine (cyan carbon atoms). LpqY is shown in cartoon format and residues are shown in grey. Dashed lines represent hydrogen bonds.

Our next goal was to identify the molecular basis of compound recognition. The hit compounds were docked to the LpqY target (PDB 7APE) using AutoDock VINA with a flexible docking protocol. The trehalose control had a score of −8.6 kJ mol^−1^ and the docked pose is identical to the binding orientation found in the LpqY-trehalose crystal structure (PDB 7APE) (Fig. S2 and S3†).^21^ Docking scores ranged between −11.1 kJ mol^−1^ and −7.3 kJ mol^−1^ (Fig. S2†). TCA1 has a higher binding score than trehalose (−10.3 kJ mol^−1^), which correlates with the increase in affinity for TCA1 compared to trehalose observed through MST. Analysis of the docked complexes indicate that each compound is predicted to bind within the trehalose pocket, Fig. 5 and S4.† The best ranked pose of TCA1, indicates that the thiophene moiety is buried in the pocket and is in close proximity to the hinge-region containing Arg404, with the carbamate group extending towards the entrance of the binding channel and the benzothiazole moiety extending into an unoccupied cavity in the LpqY-trehalose crystal structure. TCA1 is orientated to interact through three hydrogen bond interactions between its two amide groups with the side chains of Gln59 and Glu241 and the backbone of Ala56 with the benzothiazole group π-stacking with Tyr261 (Fig. 5B). Combined, the additional aromaticity of the benzothiazole moiety and the direct engagement with Ala56, which does not interact with the trehalose substrate, may explain the higher affinity of TCA1 than trehalose for LpqY. Milciclib has the most extended structure, with a length of >17 Å, and was unique in that it adopts a less buried position within the binding pocket, with the terminal amide positioned >7 Å from Arg404 (Fig. 5C). In this instance the pyrazoloquinazoline ring system superposes onto the second glucose molecule of trehalose (Fig. S4E†) with the phenylpiperazine group stretching ∼10 Å towards the binding cavity entrance. Recognition of milciclib is achieved by a mixture of polar and hydrophobic contacts. Residues Ala56, Gln59 and Glu241 form hydrogen bonds with the terminal amide group and Tyr261 is positioned ∼3.5 Å from the pyrimidine ring permitting π-stacking interactions to occur. In contrast, the thiazole group of nitazoxanide sits in a similar position to the most buried glucose molecule of trehalose, with the nitro-group directed towards Arg404 (Fig. 5D). This molecule makes polar contacts with the side chain of Asn25 and Asn134. Tyr261 and Tyr259 may also form π-stacking interactions with the phenyl and thiazole moieties of the substrate. In this position the contacts with nitazoxanide are with residues located towards the entrance of the binding cleft. The inability of nitazoxanide to engage with residues further into the binding pocket may explain its lower binding affinity compared to trehalose. In contrast, primaquine and MMV676524 bind in the trehalose binding site through recognition of the terminal amine of primaquine and the linking amino group of MMV676524 with the side-chain of Asp80 (Fig. 5E and F). Although there is some structural similarity between MMV676524 and trehalose in that both molecules contain linked ring moieties, it is clear that the more rigid and flat heterocycles, compared to puckered carbohydrate rings, prevents MMV676524 from adopting a similar orientation in the binding pocket and may explain the reduced interactions with LpqY.

Our recent high resolution structure (PDB 7APE) revealed that trehalose is co-ordinated through an extensive network of polar contracts, with all sugar hydroxy groups and the ring oxygen atom participating through interactions with Asn25, Glu26, Gln59, Asp80, Asn134, Glu41, Tyr259, Gly334 and Arg404 and hydrophobic contacts with Tyr259, Trp261 and Leu371.^21^ It is perhaps unsurprising that the hit molecules are predominantly recognised through their amide and carbonyl moieties rather than their heterocyclic rings with some of these residues, which are key determinants in mediating recognition. The hit compounds contain ≤3 hydrogen bond donors (HBDs), whereas trehalose has 8. This suggests that the total number of HBDs may be important physicochemical parameters for LpqY binding and recognition. It is possible that increasing the number of HBDs coupled with a stepwise approach to scaffold modification that incorporates transporter recognition motifs may represent an excellent starting point to obtain specific and potent leads against the LpqY-SugABC transporter. Indeed, the rational design of new antibiotics with structural modifications that enable porin permeation through bacterial membranes has been a highly effective strategy against the Gram-negative ESKAPE pathogens.^52,53^

## Conclusions

The LpqY-SugABC transporter is an attractive target for the development of new drugs as it is critical for *Mtb* to establish infection in mice and since the LpqY component is surface-exposed it may be possible to develop anti-tubercular agents that bypass the need for an antibiotic to cross the ‘impermeable’ mycobacterial cell envelope.^15,21,23^ In this work, we screened all of the drug-like compounds in the Pathogen Box for binding to the mycobacterial trehalose transporter LpqY. This resulted in the identification of five commercially available compounds with diverse scaffolds, distinct from the trehalose analogues already known to be recognised by LpqY-SugABC. Based on the proposed binding modes all hit compounds are likely to reside in the trehalose pocket. Interestingly, TCA1 has greater affinity for LpqY than trehalose suggesting that the formation of π-stacking interactions alongside polar contacts with residues buried within the trehalose pocket could be key for the development of molecules with high affinity to LpqY. To date no effective anti-tubercular agents have been designed to specifically target the LpqY-SugABC transporter either with the aim of inhibiting trehalose uptake or to facilitate uptake of bactericidal agents. Together, these results provide viable scaffold starting points to guide future drug design and with the potential to open up new avenues to treat TB.

## Conflicts of interest

There is no conflict of interest to declare.

## Supplementary Material

MD-013-D2MD00104G-s001

MD-013-D2MD00104G-s002

## References

[cit1] World Health Organisation, Global Tuberculosis Report, https://www.who.int/publications/i/item/9789240037021

[cit2] Migliori G. B., Tiberi S., Zumla A., Petersen E., Chakaya J. M., Wejse C., Munoz Torrico M., Duarte R., Alffenaar J. W., Schaaf H. S., Marais B. J., Cirillo D. M., Alagna R., Rendon A., Pontali E., Piubello A., Figueroa J., Ferlazzo G., Garcia-Basteiro A., Centis R., Visca D., D’Ambrosio L., Sotgiu G., members of the Global Tuberculosis Network (2020). Int. J. Infect. Dis..

[cit3] Batt S. M., Minnikin D. E., Besra G. S. (2020). Biochem. J..

[cit4] Brennan P. J., Nikaido H. (1995). Annu. Rev. Biochem..

[cit5] Chen F., Zhou J., Luo F., Mohammed A. B., Zhang X. L. (2007). Biochem. Biophys. Res. Commun..

[cit6] Sun X., Pan Q., Yuan C., Wang Q., Tang X. L., Ding K., Zhou X., Zhang X. L. (2016). J. Am. Chem. Soc..

[cit7] Tang X. L., Wu S. M., Xie Y., Song N., Guan Q., Yuan C., Zhou X., Zhang X. L. (2016). J. Infect..

[cit8] Guy C. S., Gibson M. I., Fullam E. (2019). Chem. Sci..

[cit9] Guy C. S., Murray K., Gibson M. I., Fullam E. (2019). Org. Biomol. Chem..

[cit10] Brecik M., Centarova I., Mukherjee R., Kolly G. S., Huszar S., Bobovska A., Kilacskova E., Mokosova V., Svetlikova Z., Sarkan M., Neres J., Kordulakova J., Cole S. T., Mikusova K. (2015). ACS Chem. Biol..

[cit11] West N. P., Cergol K. M., Xue M., Randall E. J., Britton W. J., Payne R. J. (2011). Chem. Commun..

[cit12] Chen C., Gardete S., Jansen R. S., Shetty A., Dick T., Rhee K. Y., Dartois V. (2018). Antimicrob. Agents Chemother..

[cit13] Chen H., Nyantakyi S. A., Li M., Gopal P., Aziz D. B., Yang T., Moreira W., Gengenbacher M., Dick T., Go M. L. (2018). Front. Microbiol..

[cit14] Soni D. K., Dubey S. K., Bhatnagar R. (2020). Emerging Microbes Infect..

[cit15] Fullam E., Young R. J. (2020). RSC Med. Chem..

[cit16] Cole S. T., Brosch R., Parkhill J., Garnier T., Churcher C., Harris D., Gordon S. V., Eiglmeier K., Gas S., Barry, 3rd C. E., Tekaia F., Badcock K., Basham D., Brown D., Chillingworth T., Connor R., Davies R., Devlin K., Feltwell T., Gentles S., Hamlin N., Holroyd S., Hornsby T., Jagels K., Krogh A., McLean J., Moule S., Murphy L., Oliver K., Osborne J., Quail M. A., Rajandream M. A., Rogers J., Rutter S., Seeger K., Skelton J., Squares R., Squares S., Sulston J. E., Taylor K., Whitehead S., Barrell B. G. (1998). Nature.

[cit17] Niederweis M. (2008). Microbiology.

[cit18] Braibant M., Gilot P., Content J. (2000). FEMS Microbiol. Rev..

[cit19] Li P., Gu Y., Li J., Xie L., Li X., Xie J. (2017). J. Membr. Biol..

[cit20] Fullam E., Prokes I., Futterer K., Besra G. S. (2016). Open Biol..

[cit21] Furze C. M., Delso I., Casal E., Guy C. S., Seddon C., Brown C. M., Parker H. L., Radhakrishnan A., Pacheco-Gomez R., Stansfeld P. J., Angulo J., Cameron A. D., Fullam E. (2021). J. Biol. Chem..

[cit22] Fenn J. S., Nepravishta R., Guy C. S., Harrison J., Angulo J., Cameron A. D., Fullam E. (2019). ACS Chem. Biol..

[cit23] Kalscheuer R., Weinrick B., Veeraraghavan U., Besra G. S., Jacobs, Jr. W. R. (2010). Proc. Natl. Acad. Sci. U. S. A..

[cit24] Arnold F. M., Weber M. S., Gonda I., Gallenito M. J., Adenau S., Egloff P., Zimmermann I., Hutter C. A. J., Hurlimann L. M., Peters E. E., Piel J., Meloni G., Medalia O., Seeger M. A. (2020). Nature.

[cit25] Mitra A., Ko Y. H., Cingolani G., Niederweis M. (2019). Nat. Commun..

[cit26] Bhattacharyya N., Nkumama I. N., Newland-Smith Z., Lin L. Y., Yin W., Cullen R. E., Griffiths J. S., Jarvis A. R., Price M. J., Chong P. Y., Wallis R., O’Hare H. M. (2018). MBio.

[cit27] Gopinath K., Venclovas C., Ioerger T. R., Sacchettini J. C., McKinney J. D., Mizrahi V., Warner D. F. (2013). Open Biol..

[cit28] Rodriguez G. M., Smith I. (2006). J. Bacteriol..

[cit29] Danelishvili L., Shulzhenko N., Chinison J. J. J., Babrak L., Hu J., Morgun A., Burrows G., Bermudez L. E. (2017). Antimicrob. Agents Chemother..

[cit30] Parker H. L., Tomas R. M. F., Furze C. M., Guy C. S., Fullam E. (2020). Org. Biomol. Chem..

[cit31] Swarts B. M., Holsclaw C. M., Jewett J. C., Alber M., Fox D. M., Siegrist M. S., Leary J. A., Kalscheuer R., Bertozzi C. R. (2012). J. Am. Chem. Soc..

[cit32] Backus K. M., Boshoff H. I., Barry C. S., Boutureira O., Patel M. K., D'Hooge F., Lee S. S., Via L. E., Tahlan K., Barry, 3rd C. E., Davis B. G. (2011). Nat. Chem. Biol..

[cit33] Rundell S. R., Wagar Z. L., Meints L. M., Olson C. D., O'Neill M. K., Piligian B. F., Poston A. W., Hood R. J., Woodruff P. J., Swarts B. M. (2016). Org. Biomol. Chem..

[cit34] Kamariza M., Shieh P., Ealand C. S., Peters J. S., Chu B., Rodriguez-Rivera F. P., Babu Sait M. R., Treuren W. V., Martinson N., Kalscheuer R., Kana B. D., Bertozzi C. R. (2018). Sci. Transl. Med..

[cit35] Veale C. G. L. (2019). ChemMedChem.

[cit36] Duffy S., Sykes M. L., Jones A. J., Shelper T. B., Simpson M., Lang R., Poulsen S. A., Sleebs B. E., Avery V. M. (2017). Antimicrob. Agents Chemother..

[cit37] Mayer F. L., Kronstad J. W. (2017). mSphere.

[cit38] Veale C. G. L., Hoppe H. C. (2018). MedChemComm.

[cit39] Ullah I., Gahalawat S., Booshehri L. M., Niederstrasser H., Majumdar S., Leija C., Bradford J. M., Hu B., Ready J. M., Wetzel D. M. (2020). ACS Infect. Dis..

[cit40] Mikusova K., Huang H., Yagi T., Holsters M., Vereecke D., D'Haeze W., Scherman M. S., Brennan P. J., McNeil M. R., Crick D. C. (2005). J. Bacteriol..

[cit41] Wang F., Sambandan D., Halder R., Wang J., Batt S. M., Weinrick B., Ahmad I., Yang P., Zhang Y., Kim J., Hassani M., Huszar S., Trefzer C., Ma Z., Kaneko T., Mdluli K. E., Franzblau S., Chatterjee A. K., Johnsson K., Mikusova K., Besra G. S., Futterer K., Robbins S. H., Barnes S. W., Walker J. R., Jacobs, Jr. W. R., Schultz P. G. (2013). Proc. Natl. Acad. Sci. U. S. A..

[cit42] Morris G. M., Huey R., Lindstrom W., Sanner M. F., Belew R. K., Goodsell D. S., Olson A. J. (2009). J. Comput. Chem..

[cit43] Kim S., Chen J., Cheng T., Gindulyte A., He J., He S., Li Q., Shoemaker B. A., Thiessen P. A., Yu B., Zaslavsky L., Zhang J., Bolton E. E. (2021). Nucleic Acids Res..

[cit44] Hanwell M. D., Curtis D. E., Lonie D. C., Vandermeersch T., Zurek E., Hutchison G. R. (2012). Aust. J. Chem..

[cit45] Trott O., Olson A. J. (2010). J. Comput. Chem..

[cit46] Makarov V., Manina G., Mikusova K., Mollmann U., Ryabova O., Saint-Joanis B., Dhar N., Pasca M. R., Buroni S., Lucarelli A. P., Milano A., De Rossi E., Belanova M., Bobovska A., Dianiskova P., Kordulakova J., Sala C., Fullam E., Schneider P., McKinney J. D., Brodin P., Christophe T., Waddell S., Butcher P., Albrethsen J., Rosenkrands I., Brosch R., Nandi V., Bharath S., Gaonkar S., Shandil R. K., Balasubramanian V., Balganesh T., Tyagi S., Grosset J., Riccardi G., Cole S. T. (2009). Science.

[cit47] de Carvalho L. P., Lin G., Jiang X., Nathan C. (2009). J. Med. Chem..

[cit48] Lougheed K. E., Taylor D. L., Osborne S. A., Bryans J. S., Buxton R. S. (2009). Tuberculosis.

[cit49] Pavic K., Perkovic I., Pospisilova S., Machado M., Fontinha D., Prudencio M., Jampilek J., Coffey A., Endersen L., Rimac H., Zorc B. (2018). Eur. J. Med. Chem..

[cit50] de Carvalho L. P., Darby C. M., Rhee K. Y., Nathan C. (2011). ACS Med. Chem. Lett..

[cit51] Tukulula M., Sharma R. K., Meurillon M., Mahajan A., Naran K., Warner D., Huang J., Mekonnen B., Chibale K. (2013). ACS Med. Chem. Lett..

[cit52] Durand-Reville T. F., Miller A. A., O'Donnell J. P., Wu X., Sylvester M. A., Guler S., Iyer R., Shapiro A. B., Carter N. M., Velez-Vega C., Moussa S. H., McLeod S. M., Chen A., Tanudra A. M., Zhang J., Comita-Prevoir J., Romero J. A., Huynh H., Ferguson A. D., Horanyi P. S., Mayclin S. J., Heine H. S., Drusano G. L., Cummings J. E., Slayden R. A., Tommasi R. A. (2021). Nature.

[cit53] Iyer R., Sylvester M. A., Velez-Vega C., Tommasi R., Durand-Reville T. F., Miller A. A. (2017). ACS Infect. Dis..

