# Supplementary material for: Interrogation of the Pathogen Box reveals small molecule ligands against the mycobacterial trehalose transporter LpqY-SugABC
Source: RSC Med Chem. 2022 Aug 16;13(10):1225–33. doi: 10.1039/d2md00104g (PMC9579956; doi:10.1039/d2md00104g)
Supplement: MD-013-D2MD00104G-s001 [file MD-013-D2MD00104G-s001.pdf]

Electronic Supplementary Material (ESI) for RSC Medicinal Chemistry.
This journal is © The Royal Society of Chemistry 2022

Interrogation of the Pathogen Box reveals small molecule ligands against the mycobacterial trehalose transporter LpqY-SugABC

Anjana Radhakrishnan,[‡a] Chelsea M Brown,[‡a] Collette S Guy,[‡a] Charlotte Cooper,[a]
Raul Pacheco-Gomez,[c] Phillip J Stansfeld,[ab] Elizabeth Fullam*[a]

[a] School of Life Sciences, University of Warwick, Coventry, CV4 7AL, UK
[b] Department of Chemistry, University of Warwick, CV4 7AL, UK
[c]Malvern Panalytical Ltd, Enigma Business Park, Grovewood Road, Malvern, WR14 1XZ, United Kingdom
[‡]These authors contributed equally to this study

*Email for correspondence: e.fullam@warwick.ac.uk; (Tel. +44 (0)2476 574239)

Fig. S1. Binding affinity of trehalose for LpqY. K_d measured by microscale thermophoresis (MST). Fnorm (%) is the normalised fluorescence signal of the change in MST signal. Error bars represent standard deviations from at least three independent experiments. Error bars that cannot be observed are within the symbol. Trehalose in 10 % DMSO (K_d 36.1 ± 0.5 mM).

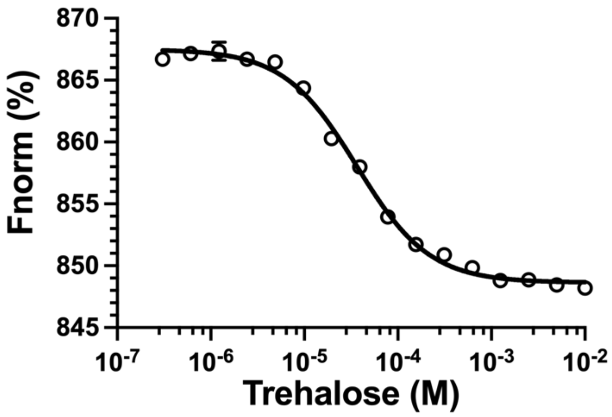

Fig. S2. Docking scores of LpqY hits. The hit compounds and trehalose were docked into the LpqY crystal structure (PDB 7APE) with AutoDock VINA. The docking score of trehalose is indicated with the dashed line.

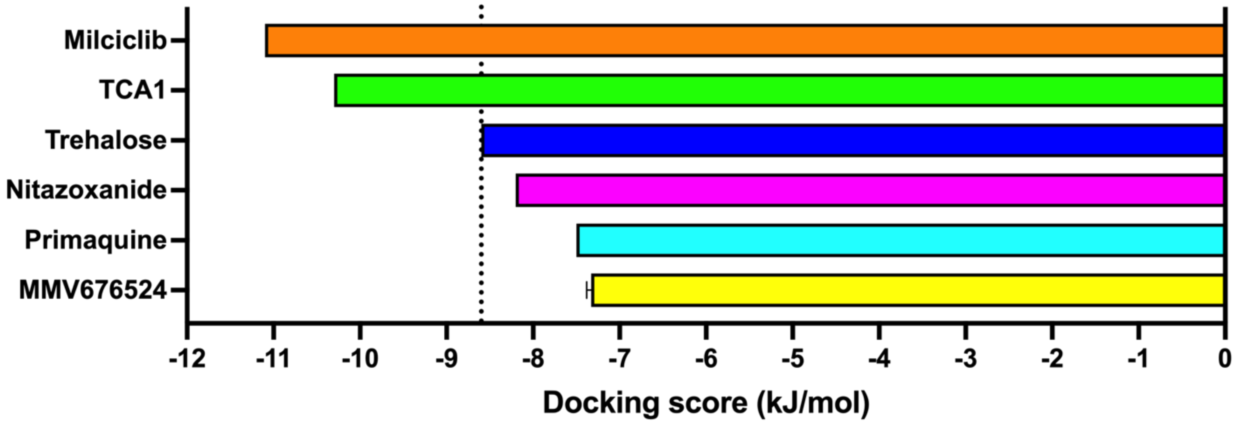

Fig. S3. Comparison of the LpqY-trehalose crystal structure with the docked pose. Illustration of the LpqY trehalose binding site showing trehalose (crystal structure PDB 7APE: green carbon atoms), docked pose (blue carbon atoms) and the interacting residues in stick representation in grey.

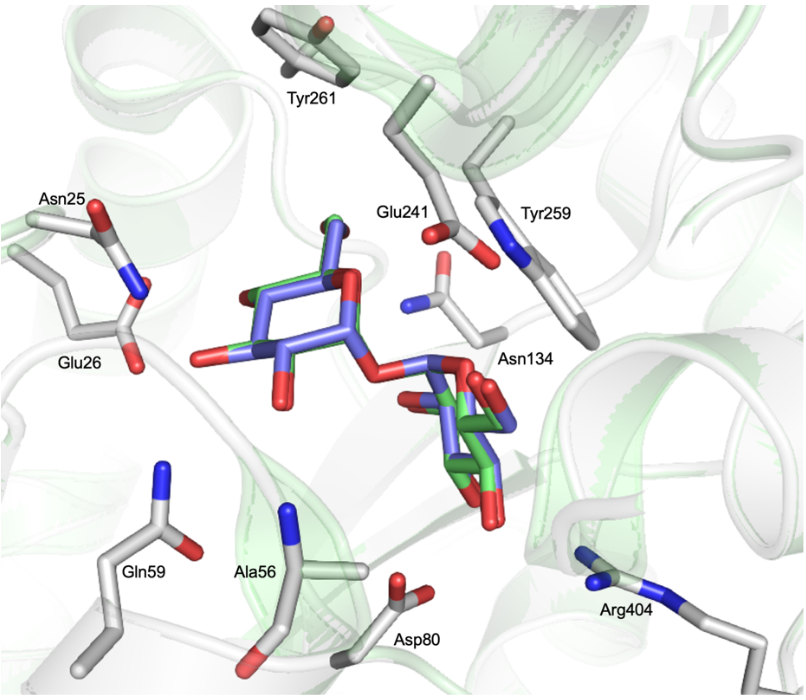

Fig. S4. Comparison of the binding site of trehalose with the docked hit compounds. Close-up illustration showing the superposition of the binding orientation of the trehalose ligand in relation to the hit compounds in stick representation. A) Overlay TCA1 (green carbon atoms), B) Nitazoxanide (magenta carbon atoms), C) MMV676524 (yellow carbon atoms), D) primaquine (cyan carbon atoms), E) milciclib (orange carbon atoms), F) Overlay of all hits with trehalose. Trehalose is illustrated with grey carbon atoms. The LpqY Arg404 is highlighted with pale blue carbon atoms.

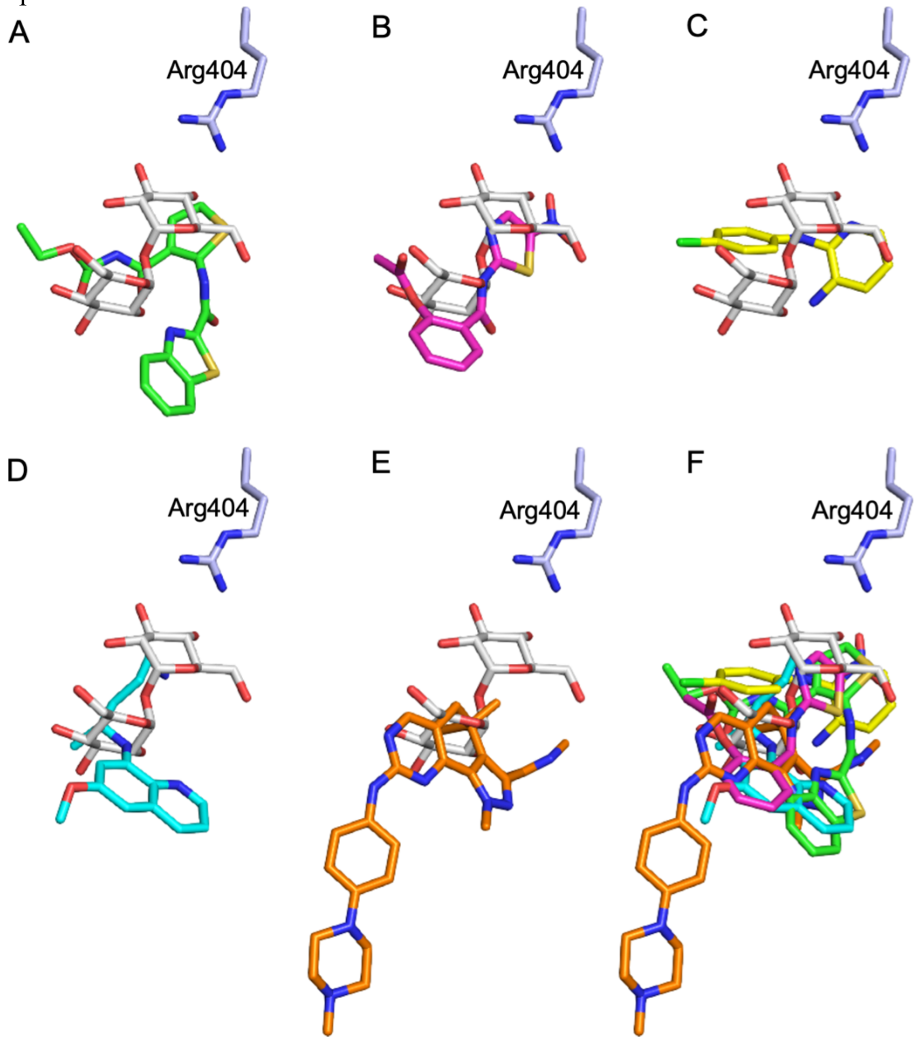

Table S1: Pathogen Box compounds with a signal to noise threshold > 5 to LpqY

Compound Number	MMV ID	Compound name	Disease set	Structure	Screen signal/noise ratio	Validation Binding score
46	MMV090930	TCA1	Tuberculosis	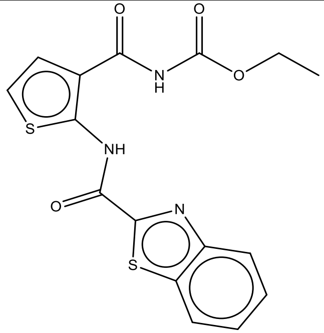	29.5	91.5
74	MMV688798	N-benzyl-2-methylsulfonyl-N-phenylbenzamide	Kinetoplastids	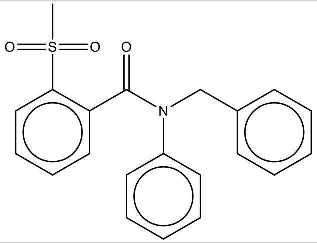	5.2	1.3
75	MMV676539	N-[2-(1H-indol-3-yl)ethyl]-2-oxochromene-3-carboxamide	Tuberculosis	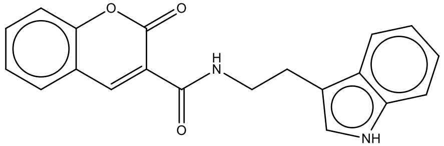	6.2	6.4
76	MMV202458	N-(4-chloro-2-methylphenyl)-2-(4-methoxyphenoxy) propanamide	Tuberculosis	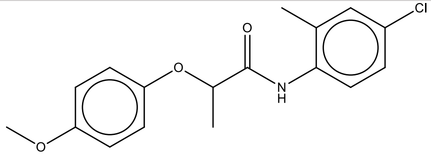	5.5	6.3
77	MMV676474	2-cyclopropyl-1-[4-(2-methoxyphenoxy) butyl]benzimidazole	Tuberculosis	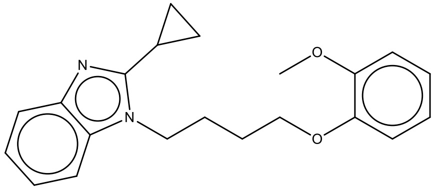	6.0	6.2

123	MMV000011	Doxycycline	Reference	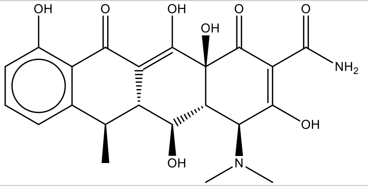	5.8	6.5
124	MMV687775	5-(3,5-dichloro-4-fluorophenyl)-3-(1-hydroxy-3,3-dimethyl-2,1-benzoxaborol-5-yl)-5-(trifluoromethyl)-4H-1,2-oxazole	Helminths	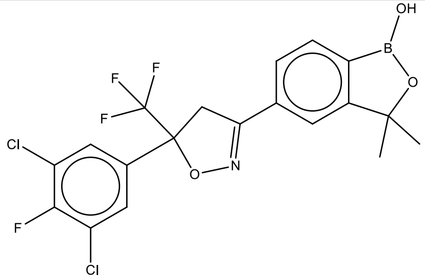	6.6	3.9
133	MMV003270	Zoxazolamine	Helminths	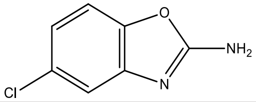	6.9	4.5
136	MMV020321	N-[2-chloro-5-(trifluoromethyl) phenyl]-2-[1-(2-methoxyphenyl) ethylamino]acetamide	Malaria	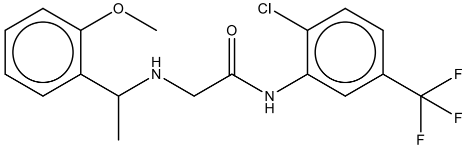	5.5	1.9
142	MMV688991	Nitazoxanide	Reference	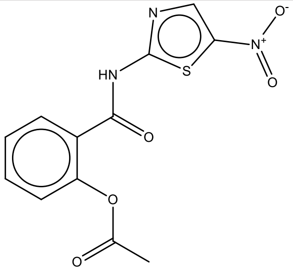	8.3	20.0
150	MMV000023	Primaquine	Reference	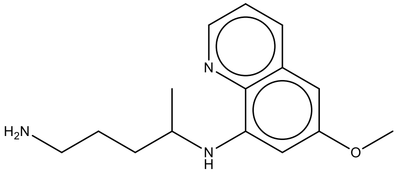	10.0	21.6

151	MMV676602	Milciclib	Kinetoplastids	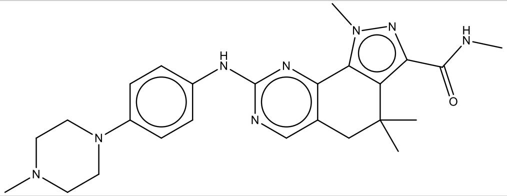	9.5	13.6
246	MMV031011	7-chloro-N-methyl-1'-(2-phenylethyl)spiro[2,3,4,9-tetrahydropyrido[3,4-b]indole-1,4'-piperidine]-3-carboxamide	Malaria	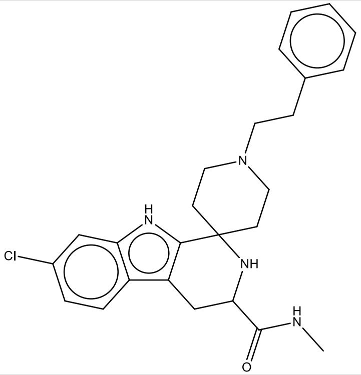	5.7	3.7
248	MMV688362	2-[4-[5-(4,5-dihydro-1H-imidazol-2-yl)-7-methoxy-1-benzofuran-2-yl]phenyl]-4,5-dihydro-1H-imidazole	Kinetoplastids	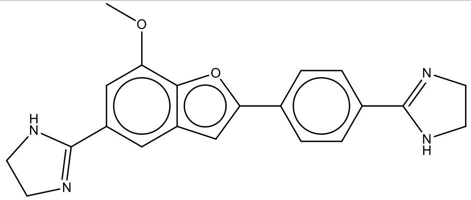	12.6	14.8
277	MMV688407	4-[4-(4,5-dihydro-1H-imidazol-2-yl)-2-methoxyphenyl]-1-[3-(4,5-dihydro-1H-imidazol-2-yl)phenyl]triazole	Kinetoplastids	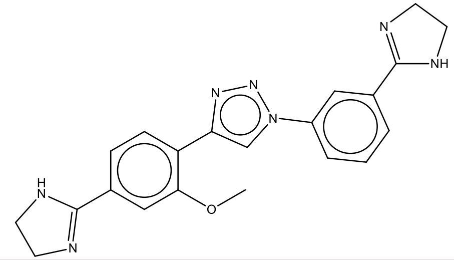	8.0	13.3
280	MMV024406	5-chloro-N-(3-chlorophenyl)-6-(4-pyridin-4-ylpiperazin-1-yl)pyridine-3-carboxamide	Malaria	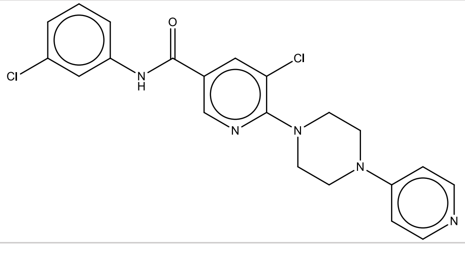	5.2	2.3

319	MMV675996	N-(4-methoxyphenyl)-N-[2-(6-methylpyridin-2-yl)ethyl] cyclohexanecarboxamide	Helminths	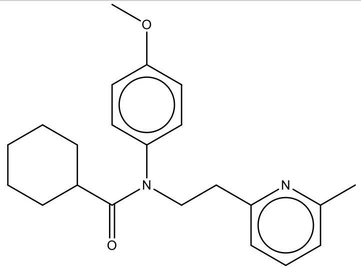	6.0	4.9
349	MMV676524	2-N-(4-chlorophenyl)pyridine-2,3-diamine	Tuberculosis	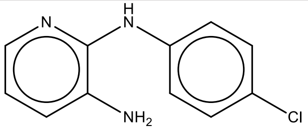	6.4	12.2
402		Trehalose	N/A	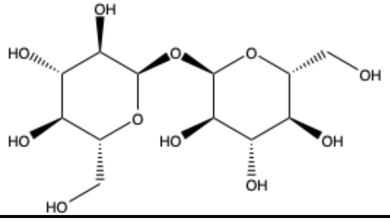	6.7	8.3

Table S2: Binding affinities for trehalose with LpqY

Compound	K_d µM
Trehalose	36.1 ± 0.5
TCA1	14.2 ± 0.8
Nitazoxanide	85.4 ± 3.4
Primaquine	ND
MMV676524	ND
Milciclib[a]	ND

Mean ± SD are from at least three independent experiments.
All assays were undertaken with 10% DMSO final concentration, apart from miciclib[a]: 20% DMSO final concentration
ND: Not able to determine K_d due to maximum solubility of the compounds (MMV676524 and milciclib) or fluorescence quenching of LpqY (primaquine).
